# A Rare Case of Quadrivalvular Infective Endocarditis

**DOI:** 10.1155/carm/6648360

**Published:** 2025-08-22

**Authors:** Mahidhar Jeedigunta, Padmakumar R., Krishnananda, Mukhyaprana Prabhu, Ashwini M. V.

**Affiliations:** ^1^Department of Cardiology, Kasturba Medical College, Manipal, Manipal Academy of Higher Education, Manipal 576104, Karnataka, India; ^2^Department of Cardiovascular Technology, Manipal College of Health Professions, Manipal Academy of Higher Education, Manipal 576104, Karnataka, India; ^3^Department of General Medicine, Kasturba Medical College, Manipal, Manipal Academy of Higher Education, Manipal 576104, Karnataka, India

**Keywords:** *Granulicatella adiacens* infective endocarditis, infective endocarditis in congenital heart diseases, quadrivalvular infective endocarditis, quadrivalvular vegetations

## Abstract

Infective endocarditis is a devastating disease with high morbidity and mortality. Infective endocarditis affecting all four valves is rarely encountered. Even rarer is the involvement of all four valves by nutritionally variant streptococci, *Granulicatella*. The case describes a female in her 40s, known case of small perimembranous ventricular septal defect, who presented with symptoms of fever and congestive cardiac failure, with severe anemia, glomerulonephritis, and pain abdomen, who was found to have vegetations on pulmonary, tricuspid, mitral, and aortic valves, with pulmonary regurgitation, tricuspid regurgitation, mitral, and aortic regurgitations. Blood culture grew *Granulicatella adiacens* species. She improved clinically after intravenous antibiotics, decongestive measures, and blood transfusion. Causation of quadrivalvular infective endocarditis is rare and previously has not been documented in *Granulicatella* infection, a fastidious species. Interestingly, the patient remained quite stable despite involvement of all four heart valves, likely due to the predominant involvement of the pulmonary valve. This case report discusses the factors predisposing to infective endocarditis in a known case of congenital heart disease and the importance of timely diagnosis and treatment.

## 1. Introduction

Infective endocarditis (IE) is a devastating disease and a great masquerader. It tests the astuteness and the patience of the clinician. Managing a case of IE requires strong clinical suspicion and even greater patience. Despite advances in the treatment of cardiovascular diseases, IE remains both highly prevalent and one of the most challenging conditions to treat. The estimated incidence of IE, as of 2019, was about 13.8 per 100,000 subjects per year. IE alone has accounted for about 66,300 deaths worldwide [1]. In case of adults with congenital heart disease, the incidence is even greater, accounting for almost double that of the general population [2]. The following case illustrates a rare presentation of IE with vegetations on all four valves in a patient with a small perimembranous ventricular septal defect (VSD).

## 2. Case Presentation

A 42-year old female, known case of acyanotic congenital heart disease, a small perimembranous VSD on medical follow-up, came with the chief complaints of pain abdomen and intermittent low-grade fever episodes, which were treated by a local physician on OPD basis for 3 months. Upon admission, the patient presented with a high-grade fever lasting 1 week, accompanied by fatigue, dyspnea, orthopnea, and bilateral pedal edema. She also reported experiencing palpitations on exertion.

On examination, she was febrile. General examination revealed poor oral hygiene with a caries tooth. Respiratory system examination showed bilateral basal end inspiratory crepitations with tachypnea. Cardiovascular examination revealed a hyperdynamic left ventricular apex. Auscultation revealed a pansystolic murmur of Grade II in the mitral area, a decrescendo early diastolic murmur in the aortic area, a Grade IV systolic murmur along with a holodiastolic murmur in the pulmonary area, and a Grade II systolic murmur in the left parasternal area. S2 was soft and had no palpable P2.

**Figure 1:**
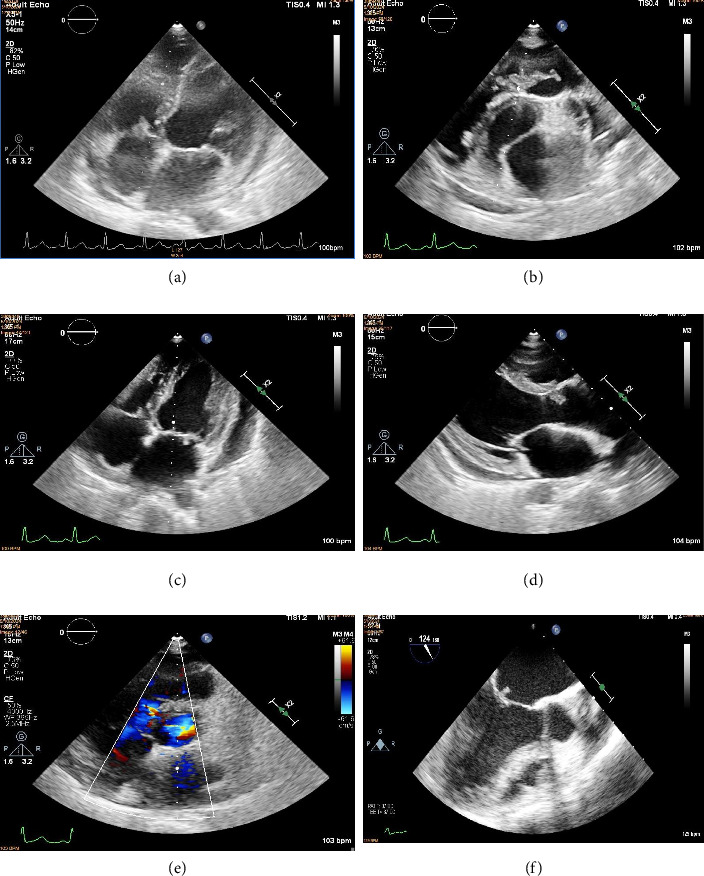
(a): Tricuspid valve vegetation, (b) pulmonary valve vegetation, (c) mitral valve vegetation, (d) aortic RCC vegetation, (e) small perimembranous VSD, and (f) mitral valve vegetation on TEE.

Her electrocardiogram revealed sinus tachycardia. Investigations revealed severe anemia of chronic disease with hemoglobin of 5 gm% and elevated creatinine of 1.7 mg/dL with microscopic hematuria. Ultrasound abdomen showed splenomegaly. A 2D echocardiogram performed showed vegetations on all four valves of the heart. The largest of the vegetations was on pulmonary valve (three vegetations measuring 12 × 6, 10 × 5, and 6 × 4 mm) with other vegetations noted on aortic right coronary cusp (RCC) (16 × 5 mm), mitral valve leaflets (7 × 5 mm), and tricuspid valve (8 × 2 mm) (Figures 1(a), 1(b), 1(c), and 1(d)). A small, restrictive perimembranous VSD of 3 mm was present causing a left-to-right shunt (Figure 1(e)). The pulmonary vegetations were seen causing severe pulmonary regurgitation, in addition to moderate aortic regurgitation, severe mitral regurgitation, and mild tricuspid regurgitation in addition to global left ventricular hypokinesia with an ejection fraction of 42%. Her autoimmune workup and investigations for immunocompromised states yielded negative results, and she had no other comorbidity predisposing to an immunocompromised state.

Her blood culture reports demonstrated the growth of nutritionally variant *Streptococcus Granulicatella adiacens* species. She was initially given blood transfusion and was started on intravenous (IV) antibiotic ceftriaxone (after sending blood samples for culture as per IE protocol), and later on, as per the sensitivity report, teicoplanin was added. Her caries tooth was treated during the course of her hospital stay. She was hemodynamically stable and improved clinically initially during the course of her hospital stay and was discharged to complete another 4 weeks of IV antibiotic therapy at a local hospital with a plan to follow her up closely for any episode of decompensation. At the end of the first hospitalization, her ejection fraction was in an improving trend (EF of 48%), with moderate aortic regurgitation, moderate mitral regurgitation, mild tricuspid regurgitation, and severe pulmonary regurgitation. She again presented with features of decompensated heart failure and fever after 10 days. Her repeat blood cultures were negative. Her echocardiogram showed no reduction in the size of the vegetations. In addition, upon further inquiry, she reported noncompliance with the prescribed diuretics. With the intent to address both the lack of response to IV antibiotic therapy and the congestion resulting from valvular regurgitation, she was empirically upgraded to vancomycin and gentamycin as per the guidelines for empirical therapy after a multidisciplinary team discussion, following which she had clinically improved. In the event of an unfavorable clinical course, had the patient not improved, early surgical intervention was designated as the contingency plan. Upfront surgery was not decided upon in view of the high risk of prosthetic valve endocarditis. She completed 6 weeks of therapy in our institution, and her repeat echocardiogram showed absent vegetation on the tricuspid valve, with small calcific nodules on the mitral, aortic, and pulmonary valves with moderate aortic and mitral insufficiency and mild pulmonic insufficiency. She was discharged on a low dose of loop diuretic (torsemide 10 mg). On follow-up, 6 months down the line, the patient is in NYHA Class I, with her latest echocardiogram showing no vegetations but damaged aortic valve cusps with moderate aortic regurgitation and currently is on the waiting list for valve replacement and VSD patch closure surgery at a government center for adult congenital heart disease.

## 3. Discussion

This case illustrates the presence of vegetations on all four valves of the heart caused by a fastidious, nutritionally variant *Streptococcus Granulicatella adiacens* species. No other previous case report of such a quadrivalvular involvement, especially with the aforementioned organism, is documented in literature.

Nutritionally variant streptococci were first described in the 1960s from the specimens of IE and were subdivided into two separate genera, *Abiotrophia* and *Granulicatella*, on the basis of their 16s rRNA sequencing [3]. Their fastidious growth demands the use of commercial medium and automated identification systems. Of these, *Granulicatella* is a known commensal of the human mouth, genital, and intestinal tracts, rarely causing infections. It is also a demonstrated cause of IE in multiple case series and reports [4]. However, quadrivalvular infection caused by the same species has not been previously documented.

*Granulicatella* growth in culture media demands special requirements such as pyridoxal and other additional agents. Three main species of *Granulicatella* have been described, *Granulicatella adiacens*, *G. elegans*, and *G. balaenopterae*. The clinical presentation of IE due to Granulicatella is similar to that of viridans group streptococci.

It is widely accepted and demonstrated that a series of interrelated events should occur prior to the formation of an infected vegetation. The initial step in the formation of an infected vegetation is injury to the valvular or cardiac endocardium, which in turn leads to the adherence of platelets and fibrin. This initially sterile platelet–fibrin plug acts as a nidus and gets secondarily infected by microorganisms during episodes of bacteremia [5]. Further activation of the coagulation system and inflammatory pathways leads to further deposition of fibronectin and eventually leads to a macroscopic excrescence or a vegetation.

Many theories are proposed for the increased incidence of vegetations in congenital and structural heart defects, including prolonged contact with blood, increased flow, and blood currents, compressive effects of abnormally directed blood streams [6]. To better explain the pathogenesis of IE, a hydrodynamic approach was proposed wherein the basic requirement for the formation of IE is that the blood should be extruded at critical high velocities through a narrow orifice into another chamber with a low pressure, aka the source-sink hypothesis [6]. The velocity of the blood in the abnormal streaming through any orifice is a function of the pressure gradient between the source and the sink. The velocity of the thus extruded blood will be greatest at a point just distal to the anatomic orifice, the vena contracta, which may form the locus of endocarditis. So, in a case of VSD, IE occurs more commonly if the orifice is small, causing a high velocity blood stream to impinge upon the endocardium of a low-pressure chamber. So, lesions generally appear on the right-sided chambers and extend into the tricuspid valve [6].

Involvement of left sided valve, especially the RCC of the aortic valve is described, but abnormal hydrodynamics due to the presence of a perimembranous VSD with a destroyed pulmonary valve due to a slow-growing bacterium may have contributed to the seeding of bacteria on all the valves causing multiple vegetations [7, 8].

In accordance with the hydrodynamics mentioned earlier, our patient had a small perimembranous VSD with a blood stream directed at the region of the pulmonary valve/right ventricular outflow tract. In accordance, the largest of the vegetations formed at that precise location on the pulmonary valve, causing severe pulmonary regurgitation. The resultant seeding of the bacteria into the right ventricle and onto the tricuspid valve is the likely reason for the formation of the tricuspid vegetation as observed along the lines of leaflet closure. Similarly, in accordance with Bernoulli's principle, due to a high velocity jet through the perimembranous VSD, there is a negative pressure created beneath the RCC. Bacteria during any episodes of bacteremia will cross the aortic valve during the systole and tend to migrate into the RCC due to the negative suction effect caused by the low-pressure zone of the high-velocity turbulent jet at the perimembranous VSD area. This likely led to the development of vegetation on the RCC. Due to the damage to the cusp and the consequent aortic regurgitation, the bacterial seeding onto the regurgitating mitral leaflets might have eventually led to the formation of a mitral vegetation.

The abovementioned case has many oddities necessitating discussion. First, even though *Granulicatella adiacens* is known to cause IE, no other case report on quadrivalvular infections has been documented. Secondly, involvement of right-sided valves is a more common phenomenon in VSD, with left-sided involvement being less common. Third, the stable state of the patient despite having such a large burden of infection is worth noting.

### 3.1. Learning Points

Although diagnosed with severe quadrivalvular IE, the patient exhibited an unexpected degree of clinical stability. Despite having quadrivalvular vegetations, only the pulmonary regurgitation was severe, with the rest being either mild or moderate. This may have been the reason for hemodynamics to be stable.

The organism being a slow-growing *Granulicatella* that caused only a subacute presentation, along with a history suggestive of partially treated endocarditis, may have contributed to her clinical stability.

This case highlights the capability of even a small VSD to cause potentially devastating endocarditis and the role of proper dental hygiene in all patients with any structural heart disease.

## Data Availability

The data that support the findings of this study are available from the corresponding author upon reasonable request. The data are not publicly available due to privacy or ethical restrictions.
